# An Electrocardiographic Clue for Pseudo-myocardial Infarction Due to Arterial Pulse–tapping Artifact: Aslanger’s Sign

**DOI:** 10.19102/icrm.2021.120904

**Published:** 2021-09-15

**Authors:** Havva Tugba Gursoy, Senay Funda Dereagzi, Ugur Caliskan, Ceren Yağmur Doğru, Furkan Kulekci, Zeynep Kaplan, Burak Bahtiyar, Andac Al, Ozcan Ozeke

**Affiliations:** ^1^Department of Cardiology, University of Health Sciences, Ankara City Hospital, Ankara, Turkey

**Keywords:** Arterial pulse–tapping artifact, electrocardiographic artifact, pseudo-myocardial infarction

## Abstract

Many factors and technical problems may alter the interpretation of electrocardiograms (ECGs). Infrequently, an artifact is considered to be the cause of ST-segment elevation, especially in asymptomatic patients. An important difference between true ST-segment elevation attributable to myocardial infarction and an artifact is that the baseline elevation in an artifact may begin before or after the onset of the QRS complex. When one encounters an abnormal ECG that exhibits suspicious wave contours and possibly only one completely normal limb lead, the diagnosis of arterial pulse artifact should be considered.

## Case presentation

**Figure 1:**
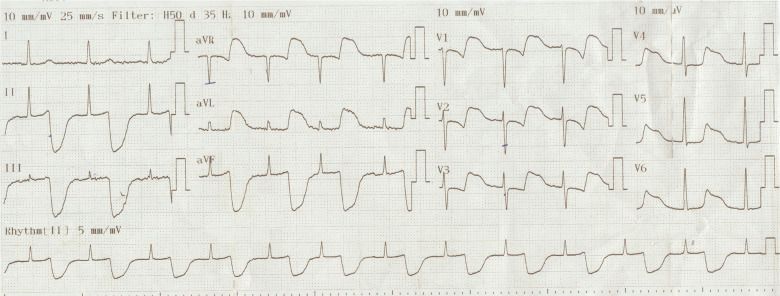
A 12-lead ECG taken at the point of first medical contact shows broad and deep T-waves with unusually abrupt onset and offset.

A 48-year-old man with suspected coronavirus disease 2019 infection was referred for primary percutaneous coronary intervention by the pre-hospital emergency system. His family history was unremarkable for cardiac disease. His functional status was class I. His physical examination revealed no abnormality. A 12-lead electrocardiogram (ECG) taken at the point of first medical contact is shown in **Figure 1**. What would be the next step for this patient?

## Discussion

Undoubtedly, many factors and technical problems may alter the interpretation of ECGs. Infrequently, an artifact is considered to be the cause of ST-segment elevation, especially in asymptomatic patients. Some common causes of baseline artifact of this nature include patient movement, a lose electrode, a dried electrode, something touching the electrode, a faulty or broken lead wire, poor skin contact due to substances on the skin, or electromagnetic interference. The electrodes should be fresh from the package and applied to skin that is clean and dry. An important difference between true ST-segment elevation attributable to myocardial infarction (MI) and an artifact is that the baseline elevation in an artifact may begin before or after the onset of the QRS complex, as was observed in this case. The broad and deep T-waves exhibited an unusually abrupt onset and offset in the current case, and the artifact was synchronous with the cardiac cycle, suggesting an arterial pulse–tapping artifact, a condition first described by Aslanger^1^ and Aslanger and Yalin,^2^ who documented a connection between similar artifacts and placement of an arm electrode on an artery.^1,2^ Sotananusak and Meemook also reported a nice demonstration of Aslanger’s sign.^3^ The timing of onset, duration, and contour of the artifact seem compatible with an arterial pulse wave altering skin contact with each heartbeat.^1,2^ The ECG diagnosis of acute MI is based on ST-segment elevation from the J point not in the middle of the ST segment as the ECG shows.

**Figure 2:**
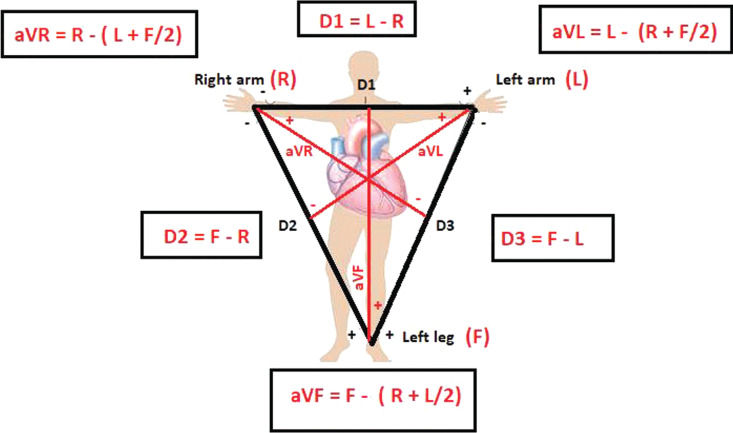
The Einthoven triangle and voltage calculations. As can be seen, the only lead that does not use the left leg electrode is lead DI. As lead DI is the only normal lead in the ECG in **Figure 1**, the left leg electrode must be the affected electrode.

ECG measures the voltage between two points on the body surface. A lead in ECG is the voltage difference between two points on the body. According to the Einthoven triangle, leads I, II, and III are bipolar limb leads. Lead I is found by comparing electric differences between the right and the left arms, lead II is found by comparing the right arm and the left leg, and lead III is found by comparing the left arm and the left leg **(Figure 2)**. Indeed, most of the electrocardiography devices record leads I and II and then calculate other limb derivations from these two derivations.^1^ The augmented limb leads that use the Goldberger central terminal, an averaging of inputs from two of the three limb electrodes, as their negative pole were also abnormal. When one of the limb electrodes is affected by a source of disturbance, it distorts not only the corresponding derivation but also leads III, aVR, aVL, and aVF, which are all calculated by mathematical equations from the first two derivations, including the distorted one **(Figure 2)**. Therefore, if the left arm electrode is affected, the only normal limb derivation will be lead II. If the left leg electrode is affected, the only normal limb derivation will be lead I. If the right arm electrode is affected, either lead III will be minimally affected or there will be no unaffected limb lead.^1,2^ Moreover, limb artifacts also affect precordial leads due to the Wilson central terminal, which constitutes the negative pole of the unipolar leads, and is produced by connecting three limb electrodes via a simple, resistive network to give an average potential across the body. Therefore, there may be only one, if any, unaffected lead in the entire spectrum of 12 derivations.^4^

In the current ECG, there was a clue to identify the problematic electrode from the ECG. The appearance of artifact occurred in all the leads except lead I. The artifact is in the bipolar extremity leads and was only seen in leads involving the left leg (leads II and III), and the amplitude of the artifact is identical in leads II, III, and aVF, whereas the amplitudes in leads aVR and aVL are only half the size. In the precordial leads, the amplitude is smaller but identical in all six leads. When one encounters an abnormal ECG that exhibits suspicious wave contours and possibly only one completely normal limb lead, the diagnosis of arterial pulse artifact should be called to mind.
